# Homozygosity for the *C9orf72* GGGGCC repeat expansion in frontotemporal dementia

**DOI:** 10.1007/s00401-013-1147-0

**Published:** 2013-07-02

**Authors:** Pietro Fratta, Mark Poulter, Tammaryn Lashley, Jonathan D. Rohrer, James M. Polke, Jon Beck, Natalie Ryan, Davina Hensman, Sarah Mizielinska, Adrian J. Waite, Mang-Ching Lai, Tania F. Gendron, Leonard Petrucelli, Elizabeth M. C. Fisher, Tamas Revesz, Jason D. Warren, John Collinge, Adrian M. Isaacs, Simon Mead

**Affiliations:** 1 Department of Neurodegenerative Disease, UCL Institute of Neurology, Queen Square, London, WC1N 3BG UK; 2 Queen Square Brain Bank, Department of Molecular Neuroscience, UCL Institute of Neurology, Queen Square, London, WC1N 3BG UK; 3 Dementia Research Centre, Department of Neurodegenerative Disease, UCL Institute of Neurology, Queen Square, London, WC1N 3BG UK; 4 MRC Prion Unit, Department of Neurodegenerative Disease, UCL Institute of Neurology, Queen Square, London, WC1N 3BG UK; 6 Neurogenetics Unit, National Hospital for Neurology and Neurosurgery, Queen Square, London, WC1N 3BG UK; 7 Institute of Psychological Medicine and Clinical Neurosciences, MRC Centre for Neuropsychiatric Genetics and Genomics, School of Medicine, Cardiff University, Cardiff, CF14 4XN UK; 8 Department of Neuroscience, Mayo Clinic Florida, Jacksonville, FL 32224 USA

**Keywords:** C9orf72, ALS, FTD

## Abstract

An expanded hexanucleotide repeat in the *C9orf72* gene is the most common genetic cause of frontotemporal dementia and amyotrophic lateral sclerosis (c9FTD/ALS). We now report the first description of a homozygous patient and compare it to a series of heterozygous cases. The patient developed early-onset frontotemporal dementia without additional features. Neuropathological analysis showed c9FTD/ALS characteristics, with abundant p62-positive inclusions in the frontal and temporal cortices, hippocampus and cerebellum, as well as less abundant TDP-43-positive inclusions. Overall, the clinical and pathological features were severe, but did not fall outside the usual disease spectrum. Quantification of C9orf72 transcript levels in post-mortem brain demonstrated expression of all known C9orf72 transcript variants, but at a reduced level. The pathogenic mechanisms by which the hexanucleotide repeat expansion causes disease are unclear and both gain- and loss-of-function mechanisms may play a role. Our data support a gain-of-function mechanism as pure homozygous loss of function would be expected to lead to a more severe, or completely different clinical phenotype to the one described here, which falls within the usual range. Our findings have implications for genetic counselling, highlighting the need to use genetic tests that distinguish *C9orf72* homozygosity.

**Electronic supplementary material:**

The online version of this article (doi:10.1007/s00401-013-1147-0) contains supplementary material, which is available to authorized users.

## Introduction

Frontotemporal dementia (FTD) is the most common form of early-onset dementia, second only to Alzheimer’s disease, and is characterised clinically by changes in personality or language impairment and pathologically by the progressive degeneration of the frontal and anterior temporal lobes [21, 24]. FTD shares numerous similarities at the genetic and neuropathological level with amyotrophic lateral sclerosis (ALS), a devastating neurodegenerative disorder in which the loss of motor neurons in brain and spinal cord causes progressive weakness and paralysis, ultimately leading to death from respiratory failure [12]. ALS and FTD can co-occur, and they have been proposed to be part of the same spectrum of disease [17].

An expanded hexanucleotide repeat in the *C9orf72* gene has been identified recently as the most common known genetic cause of both FTD and ALS [9, 20, 25]. Whilst <33 hexanucleotide repeats occur in the healthy general population, with just 2 repeats being the most common form, *C9orf72* ALS/FTD cases carry 800–4,400 repeats [5]. *C9orf72* positive FTD (c9FTD) cases may show clinically typical FTD features and have been described to most commonly present with behavioural variant frontotemporal dementia, often with prominent psychiatric and amnestic symptoms [19].

Pathologically, c9FTD patients have unique characteristics, including p62-positive neuronal cytoplasmic inclusions (NCIs) in cerebellar and dentate fascia granule cells and pyramidal neurons of the hippocampus [19, 23, 26]. The pathogenic mechanisms by which the hexanucleotide repeat expansion causes disease are unclear and both gain- and loss-of-function mechanisms have been proposed to play a role [3, 9, 10, 22]. Here, we present a case of FTD with a homozygous *C9orf72* hexanucleotide repeat expansion and compare with heterozygous cases. Clinical features, neuropathology and expression data that we describe below carry important implications for disease pathogenesis and genetic counselling.

## Materials and methods

### DNA extraction and genotyping

Genomic DNA was extracted from peripheral blood using the Nucleon BACC2 DNA extraction kit (RPN8502) following the supplied protocol. DNA concentrations were determined using a Nanodrop ND-1000 spectrophotometer, and adjusted to a working concentration of 20 ng/μl TE buffer.

Rs3849942 genotyping: The surrogate marker rs3849942, defining the haplotypes at risk of expansion, was genotyped by allelic discrimination using the 5′ nuclease assay in conjunction with Minor Groove Binding (MGB) probes. The custom-designed assay was performed on the SDS7500 Fast Real Time PCR system (ABI) and genotyping calls were made using software v2.0.6.

### Hexanucleotide repeat number assessment

Hexanucleotide repeat number was assessed by repeat primed PCR and carried out as previously described [19]. Fragment length analysis was undertaken on an ABI 3730xl automated sequencer. Analysis of repeat primed PCR (rpPCR) electrophoretograms was performed using Peak Scanner v1.0 (ABI). In addition, repeat number was assessed by fluorescent labelled PCR followed by fragment length analysis on an Applied Biosystems (ABI) 3730xl automated sequencer as previously described [19].

### Microsatellite analysis

Microsatellite analysis was performed using ten markers spanning approximately 13.1 Mb of genomic DNA centred on the C9orf72 gene. PCR amplicons were generated using fluorescently end-labelled primers for microsatellite markers D9S1814(VIC), D9S976(FAM), D9S171(NED), D9S1121(VIC), D9S169(FAM), D9S263(HEX), D9S270(FAM), D9S104(FAM), D9S147E(NED) and D9S761(FAM). DNA products were electrophoresed on an ABI 3730xl automated sequencer. Data were analysed using ABI GeneMapper software v4.0 [Applied Biosystems (ABI)].

### Southern blotting

Adaptation of standard blotting methods included the probing of AluI/DdeI digested genomic DNA with an oligonucleotide hybridisation probe from Eurofins MWG Operon (Germany) that comprised five hexanucleotide repeats (GGGGCC)_5_ labelled 3′ and 5′ with digoxigenin (DIG). Further methods followed the DIG Application Manual [Roche Applied Science (RAS)], except for the supplementation of DIG Easy Hyb buffer with 100 μg/ml denatured fragmented salmon sperm DNA. Detection was carried out as recommended in the DIG Application Manual using CSPD ready-to-use (RAS) as chemiluminescent substrate visualised on fluorescent detection film (RAS). Hexanucleotide repeat number was estimated by visual interpolation using a plot of log10 base pair number against migration distance which was created in Excel (Microsoft) and subtraction of the wild-type allele fragment size (199 bp). Full methods have been previously reported [5].

For single probe Southern hybridisation, 5 μg genomic DNA were digested with *Eco*RI. The Roche DIG labelling and detection system was used with a 1 kb *C9orf72* genomic DNA specific probe.

### Pathological analysis

Analysis was undertaken according to the Queen Square Brain Bank protocol [16]. p62 (1:200, BD Transduction Laboratories), TDP-43 (amino acids 1-261, Abnova, 1:800), C9RANT [3] and ubiquilin-2 [6] were analysed. Using a semi-quantitative approach, the frequency of p62-positive neuronal cytoplasmic inclusions was compared with TDP-43-positive inclusions in the reported regions of the frontal cortex, temporal cortex, hippocampus and cerebellum.

Semi-quantitative analysis of p62 and TDP-43-positive lesions was performed using a five-tiered semi-quantitative grading scale. The scoring of the pathological lesions was as follows: ‘0’ for the absence of p62-positive neuronal cytoplasmic inclusions (NCIs) and neuronal intranuclear inclusions (NIIs); ‘+’ for 1–5 inclusions present in an average of at least five microscopic fields using a 20× objective; ‘++’ for 6–10; ‘+++’ for between 11 and 20 inclusions; ‘++++’ for >20 lesions.

### RNA extraction and transcription analysis

RNA extraction was performed using the miRNeasy Kit (Qiagen) and RNA quality was evaluated using the Agilent 2100 Bioanalyzer. Reverse transcription was performed using the QuantiTect kit (Qiagen) and real-time quantitative RT-PCR (qPCR) was performed using Taqman probes according to manufacturer’s instructions (Applied Biosytems Primers and probes were designed in order to specifically detect the V1, V2 and V3 *C9orf72* isoforms (Supplementary table 1**)**. Dilution curve experiments using the V1 and V3 *C9orf72* primers vs beta-actin showed comparable efficiencies. Reactions were performed in triplicate and gene expression values were normalised using the housekeeping genes *ACTB* and *GAPDH*. Relative quantification of gene expression was calculated via the comparative threshold cycle (ddCt) method. Regression analysis was performed by plotting the expression values vs the number of mutated alleles using Graphpad Prism v5.03.

## Results

### Patient report

A 45-year-old man originally from Turkey presented to a cognitive disorders clinic with a 2-year history of change in behaviour and cognitive impairment. He had become apathetic with increasing irritability and his behaviour was described as having become increasingly childish. Over the following 2 years prior to presentation he rapidly deteriorated such that when assessed he was severely apathetic and speech output was minimal. He was unable to follow more than simple commands and did not recognise his family. He required assistance with dressing and eating and was doubly incontinent.

**Fig. 1:**
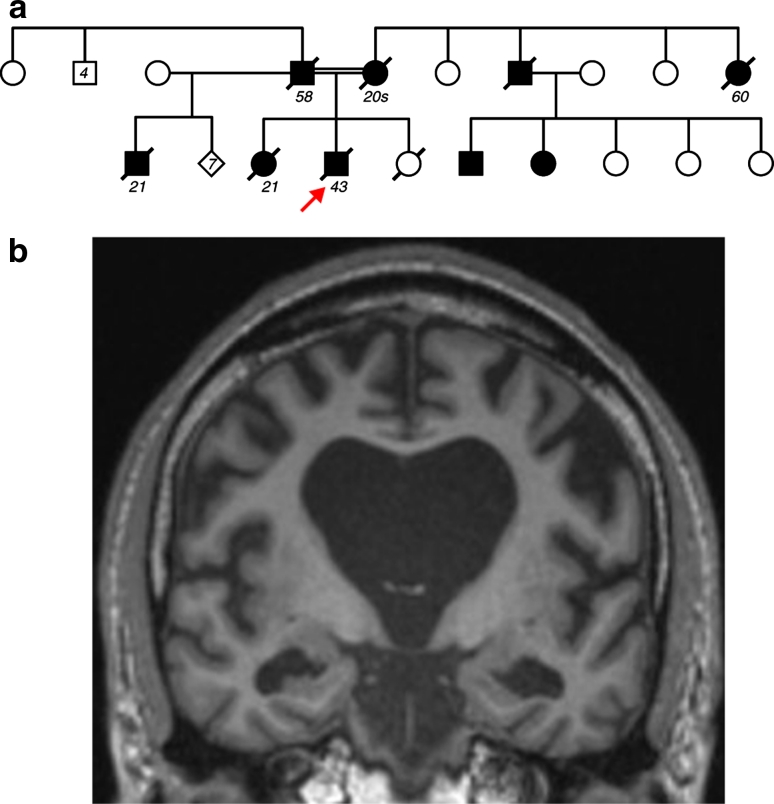
Family history and clinical features. **a** Pedigree of the family. *Arrow* identifies the proband; age of onset is indicated below affected individuals (*filled symbols*). **b** Coronal brain MRI T1-weighted scan, showing bilateral widespread atrophy

The proband’s mother and father were first cousins, their fathers being brothers. Both parents developed an early-onset dementia, and a number of other family members developed dementia, with an autosomal dominant compatible transmission (Fig. 1a).

Neurological examination revealed an MMSE of 9 and relatively global cognitive impairment with formal neuropsychometric testing. The proband was echolalic. During testing he was perseverative and disinhibited. He had a mild extrapyramidal syndrome with bilateral limb bradykinesia and rigidity with stimulus-sensitive myoclonus and limb apraxia. He had symmetrically brisk reflexes with downgoing plantar responses. Limb power and co-ordination were normal with no wasting or fasciculations—EMG was not performed. He continued to progress rapidly with increasingly disinhibited and aggressive behaviour and died less than a year later.

Cerebro-spinal fluid (CSF) examination revealed tau levels of 171 pg/ml (0–320) and Aβ1-42 of 180 pg/ml (220–2,000). Oligoclonal bands were negative and CSF was acellular. EEG was unremarkable. A brain MRI showed generalised atrophy with frontal and medial temporal predominance without any clear asymmetry (Fig. 1b).

### Genetic analysis

**Fig. 2:**
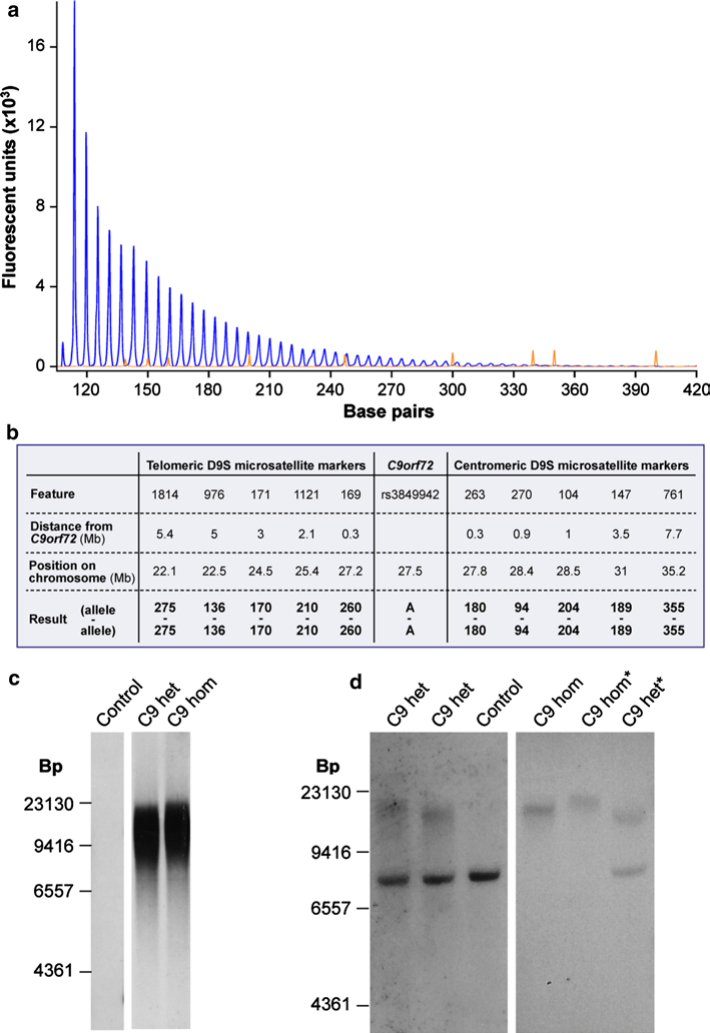
Presence of a homozygous hexanucleotide expansion in *C9orf72*. **a** Results of repeat primed PCR for *C9orf72* expansion demonstrating the saw-tooth pattern, typical of the pathological expansion. Repeats are measurable up to 40 hexanucleotide repeats. The size of fluorescently labelled DNA amplicons is shown in base pairs (bp) against the expected asymptotic decay in fluorescence measured in arbitrary units. The GS500 size standard can also be seen with* red peaks* at 300, 340, 350, 400, 450, 490 and 500 bp. **b** Results of genetic analysis of the proband that demonstrate homozygosity at all microsatellite marker positions and at rs3849942. Positions of markers are given in megabases relative to chromosome and *C9orf72* position. **c** Southern blot analysis on blood-derived DNA using a probe directed to the hexanucleotide repeat (GGGGCC)_5_ shows the presence of an expansion in the proband (C9 hom). The expansion is calculated to have a maximum of 3,067, a minimum 830 and mode of 2,297 repeats and is indistinguishable from a *C9orf72* heterozygous case (C9 het). **d** Southern blot analysis on blood and brain (*asterisk*) derived DNA using a single-copy probe detecting the sequence adjacent to the repeat. The normal allele runs as an 8-kb band and is detectable in controls and *C9orf72* expansion heterozygous cases, but is absent in the proband (C9 hom)

The patient’s DNA was sequenced to screen the *PRNP*, *APP*, *PSEN1*, *PSEN2*, *GRN*, and *MAPT*, genes, all of which were negative for mutations, and for *C9orf72* hexanucleotide repeat number by repeat primed PCR, which showed a minimum of 40 repeats and a positive expansion pattern (Fig. 2a). The presence of a large expansion was confirmed by Southern blotting on DNA from peripheral lymphocytes using a GGGGCC_5_ oligonucleotide probe that targets the expansion [5], revealing a maximum of 3,067 repeats, minimum 830 (mode of 2,297 repeats, Fig. 2c). Assessment of repeat number of the presumed non-expanded allele by fluorescent PCR appeared to fail repeatedly, not revealing any bands despite test sensitivity proven from 2 to 32 repeats [5]. Results of haplotype analysis using 10 highly polymorphic microsatellite markers spanning 13.1 Mb centred on *C9orf72* showed homozygosity at all markers and genotyping of rs3849942 demonstrated homozygosity for the adenosine nucleotide at this polymorphic site, typically linked with expanded alleles (Fig. 2b). These results pointed to the presence of a homozygous *C9orf72* expansion and indeed, Southern blotting performed using a single-copy probe detecting the sequence adjacent to the repeat showed lack of a normal allele, confirming homozygosity of the *C9orf72* expansion (Fig. 2d).

### Neuropathology analysis of C9orf72 homozygous and heterozygous cases

**Fig. 3:**
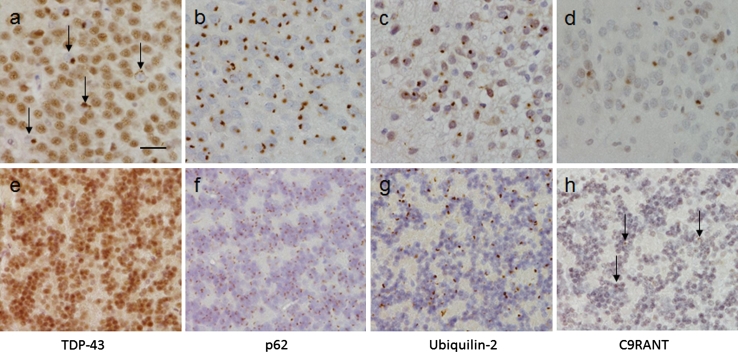
Immunohistochemical analysis of the neuronal cytoplasmic inclusions in the *C9orf72* homozygous case. The ‘*star-like*’ neuronal cytoplasmic inclusions observed in the granule cell layer of the hippocampus (**a**–**d**) and the cerebellum (**e**–**h**) have been shown to be positive to varying degrees with several antibodies to proteins shown to be associated with *C9orf72* cases. TDP-43 immunohistochemistry showed compact neuronal cytoplasmic inclusions (**a** *arrows*) together with the normal neuronal nuclear staining pattern. Only the normal nuclear staining pattern was observed in the cerebellum with the TDP-43 antibody (**e**). p62 immunohistochemisty (**b** and **f**) demonstrated large numbers of ‘*star-like*’ inclusions in both the GCL (**b**) and cerebellum (**f**). Ubiquilin-2 has been shown to be present in the small ‘*star-like*’ inclusions in heterozygous *C9orf72* cases. The homozygous presented here also demonstrated a similar staining pattern with inclusions found in the GCL (**c**) and cerebellum (**g**). The newly identified C9RANT protein was also shown to be present in a small proportion of inclusions in the GCL (**d**) and cerebellum (**h** *arrows*). *Bar* in **a** represents 20 μm in all panels

**Fig. 4:**
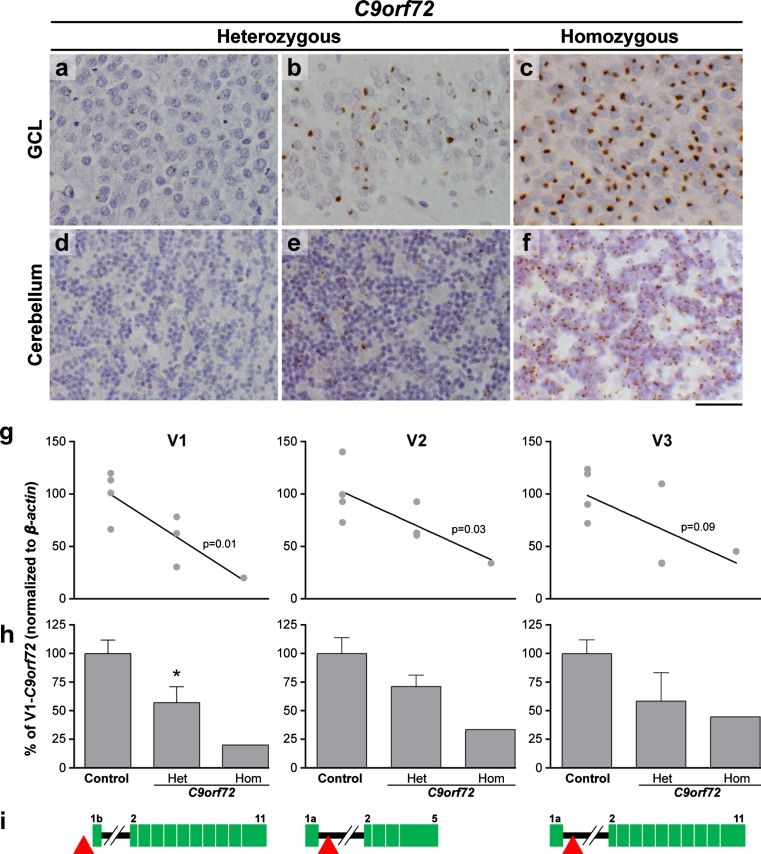
Histopathological features and *C9orf72* expression. p62 immunohistochemistry in the granule cell layer (*GCL*) of the hippocampus (**a**–**c**) and cerebellum (**d**–**f**) demonstrating the number of inclusions observed in the heterozygous cases range from mild (**a**, **d**) to severe (**b**, **e**). The severity of the inclusions observed in the homozygous case (**c**, **f**) is shown in both the GCL and cerebellum. *Bar* represents 100 μm in all panels. Real-time quantitative RT-PCR expression analysis of the three known *C9orf72* isoforms—V1, V2, V3 (**g**, **h**)—schematically represented in **i**. Hexanucleotide expansion (*red triangle*), exons (*green*), intron (*black line*). Linear regression analysis (**g**) between expression and number of normal alleles shows significance for V1 and V2. Reduction of expression in *C9orf72* heterozygous cases is significant only for V1 (*p* = 0.03)

Neuropathological examination revealed a reduced brain weight (1,015 g), with frontoparietal atrophy. The corpus callosum, caudate, globus pallidus, thalamus, amygdala and hippocampus were all reduced in bulk. There was also pallor of the substantia nigra and locus coeruleus. However, the cerebellar white matter and dentate nucleus appeared normal. Microscopic assessment identified morphological features consistent with FTLD-TDP type A including TDP-43-positive neuronal cytoplasmic inclusions (NCIs), neuronal intranuclear inclusions (NIIs) and short neuritic threads. TDP-43-positive neurites and oligodendroglial inclusions were found in the deep white matter of the frontal cortex (Supplementary figure 1). No spinal cord was available for study; however, motor neurons of the 7th and 12th nerve nuclei were available for pathological assessment and showed no TDP-43 or p62-positive inclusions. There was no evidence of hippocampal sclerosis, whilst AT8 immunohistochemistry showed neuropil threads and neurofibrillary tangles in the transentorhinal and entorhinal cortices in keeping with Braak and Braak stage II. p62-positive NCIs were evident not only in the hippocampus and cerebellum, a consistent feature of *C9orf72* expansion cases, but were also present in the majority of neurons in the middle and deeper cortical layers (Table 1; Fig. 3b, f). In keeping with previous descriptions of *C9orf72* expansion cases ubiquilin-2 inclusions were found in both cerebellum and hippocampus (Fig. 3c, g). Further, we investigated the presence of dipeptides derived from the unconventional translation of the hexanucleotide repeat and found these to be present in hippocampal and cerebellar neurons (Fig. 3d, h). In all regions the number of p62-positive NCIs outnumbered the TDP-43 pathology. The p62 and TDP43 pathology was compared to five C9orf72 heterozygous cases and assessed as severe but within the range of *C9orf72* cases (Table 1; Fig. 4a).

**Table 1:** Semi-quantitative analysis of p62 and TDP-43-positive lesions observed in heterozygous (cases 1–5) and homozygous (case 6) *C9orf72* cases

Anatomical region	Case number											
	1: Het		2: Het		3: Het		4: Het		5: Het		6: Hom	
	TDP-43	p62	TDP-43	p62	TDP-43	p62	TDP-43	p62	TDP-43	p62	TDP-43	p62
Frontal cortex												
Grey matter	++	+	++	++	+++	+++	+	++	++	+++	+	++++
White matter	++	++	++	++	++	++	0	0	0	0	+	++
Temporal cortex												
Grey matter	+	++	+	++	++	+++	+	++	++	+++	+	++++
White matter	+	+	+	+	+	++	0	+	0	+	+	++
Hippocampus												
GCL	+++	++++	+	+++	++	++++	+++	+++	+	+++	++	++++
CA1	+	+++	+	++	0	++++	+	+++	0	+++	+	++++
CA2	0	+++	0	++	0	+++	+	+++	0	+++	0	++++
CA3	0	+++	0	++	0	+++	+	+++	0	+++	0	++++
CA4	0	+++	0	+++	0	+++	–	+++	0	+++	+	++++
Subiculum	+	++	+	+	+	++	+	++	0	++	+	++++
Entorhinal	+	++	++	++	+	+++	+	++	+	++	+	++++
Fusiform	+	++	++	++	++	+++	+	++	+	++	+	++++
Cerebellar white matter	0	+	0	++	0	+++	0	+++	0	++	0	+++
Cerebellar GCL	0	++	0	++	0	++++	0	+++	0	+++	0	++++

The severity of p62 and TDP-43-positive pathology was evaluated in the homozygous and the heterozygous *C9orf72* cases. A five-tiered semi-quantitative grading scale was used in which the pathological lesions were scored as ‘0’ describing the absence of p62-positive neuronal cytoplasmic inclusions (NCIs) and neuronal intranuclear inclusions (NIIs), score ‘+’ corresponded to 1–5 inclusions present in an average of at least five microscopic fields using a 20× objective, score ‘++’ was given if the number of lesions was 6–10 while score ‘+++’ was given when the number of inclusions was between 11 and 20. Score ‘++++’ corresponded to >20 lesions

### C9orf72 transcription analysis of C9orf72 isoforms

For quantitative RT-PCR analysis for the three known *C9orf72* transcripts [9], we extracted RNA from the frontal cortex of the proband, three heterozygous *C9orf72* FTD cases and four pathologically confirmed controls without neurodegenerative disease. Results, normalised to *β*-*actin*, showed a significant reduction to 57 % of transcript variant 1 (V1) in heterozygous cases versus controls, whereas the homozygous case was reduced further to 20 % of control expression. Transcript variants 2 and 3 (V2 and V3) also showed a reduction in heterozygous cases, although not statistically significant, and a further reduction, respectively, to 34 and 45 % in the homozygous case versus controls (Fig. 3b, c). Results were confirmed using *GAPDH* as a second endogenous control.

## Discussion

We report the first FTD patient carrying a homozygous *C9orf72* hexanucleotide repeat expansion. Our finding that the clinicopathological features were severe, but within the range of reported heterozygous cases, suggests that the condition is truly dominant; therefore, we provide evidence in favour of a gain-of-function mechanism. Whilst the affected sister with an age of onset of 21 could also be potentially homozygous for the *C9orf72* expansion, it is highly unlikely that the patient’s mother and also the father’s son from his second (non-consanguineous) marriage, both with an onset in their 20s, were homozygous. These family members were not available for assessment.

We report Southern blotting for detection of *C9orf72* expansions using two different approaches with the probe directed towards either the hexanucleotide repeat itself (Fig. 2c) or the adjacent genomic region (Fig. 2d). The former has the advantage of being more sensitive and is able to detect a significant amount of somatic mosaicism, whilst the latter has the advantage of detecting also the normal allele and therefore its absence in the setting of homozygosity. A size shift, possibly due to somatic instability, is documented between blood and brain DNA samples, as previously reported [5].

Three major *C9orf72* transcripts have been described: V1, in which the hexanucleotide expansion is in the promoter region, and V2 and V3, in which the expansion lies in the first intron and is therefore transcribed [9]. Previous analyses in *C9orf72* heterozygous cases showed a reduction in V1 and V2 [9, 10]. Consistent with these findings, our results indicate a reduction of all three transcripts in heterozygous cases, with a further reduction in the homozygous case; but importantly, we unequivocally show that transcripts derived from expansion-containing alleles are present and clearly detectable in the homozygous case. One study [9] observed a greater reduction in the V1 isoform than V2 and V3. Although our results show that all variants are reduced, we nonetheless observe a greater reduction in V1 compared to V2 and V3 in the homozygous case.

The pathogenic mechanism of the *C9orf72* repeat expansion is unknown and both loss of function (LOF) [10] and gain of function [22] have been proposed to play a role. Although the two mechanisms are not mutually exclusive, the clinicopathological data along with the expression profile of the case presented here are consistent with a predominant gain-of-function mechanism. A pure LOF mutation would be expected to cause a much more severe clinical presentation and pathology in the homozygous state, which was not observed in this case. Indeed heterozygous LOF mutations in other neurodegenerative diseases present with radically different phenotypes when homozygous, whereas those associated with a toxic gain of function tend to be present with the same clinical syndrome, although in many cases with an earlier onset. Examples include heterozygous LOF mutations in the progranulin gene (*PGRN*) causing FTD but homozygous mutations causing a lysosomal storage disorder [4, 29]; mutations in *GBA* causing Parkinson’s disease in heterozygosity and Gaucher’s disease in homozygosity [1]; and mutations in *TREM2* causing Alzheimer’s disease and Nasu–Hakola disease [11, 14]; however, homozygous mutations in gain-of-function neurodegenerative diseases such as inherited prion disease (*PRNP*), ALS (*SOD1*) and Huntington disease (*HTT*), manifest with severe phenotypes, that fall within the range of disease [13, 15, 28, 30]. Further, a severe form of disease, but with no additional clinical features, such as presented here, was described for homozygous cases of myotonic dystrophy types 1 and 2 (DM1 and DM2) [2, 27]. Importantly, these disorders are similar to *c9FTD* in that they are also caused by very long expansions of non-coding repeats, and functional evidence suggests they occur via a gain-of-function mechanism [7]. The severe pathology here reported in the C9orf72 homozygous case could be compatible with a dosage dependence of the GOF mechanism. Gain and loss of function have been shown to coexist in other neurodegenerative disorders [8, 18, 31, 32]. Given the decreased levels of all *C9orf72* transcripts, further work is needed to assess the possible role of loss of function in *C9orf72* ALS/FTD, but our results argue against a pure loss-of-function mechanism.

Lastly, *C9orf72* is estimated to have a frequency of 1:700 in the UK population [5], thus making the predicted frequency of homozygous cases to be approximately 1:2 × 10^6^. Although we present a single case and clinical variability is likely to occur, our results show that homozygous *C9orf72* expansion is viable and that it can present as typical FTD. Genetic diagnostic tests are already available for *C9orf72* and are carried out by performing both repeat-primed PCR and Southern blotting. Our results underline the importance of performing Southern blots in the diagnostic test, in order to detect potential homozygous cases. Although homozygosity may or may not have prognostic implications, it carries huge implications for genetic counselling. In summary, we provide the first report of a homozygous *C9orf72* repeat expansion FTD case, which provides new insight into disease pathogenesis and important implications for genetic testing.

## Electronic supplementary material

Below is the link to the electronic supplementary material.

Supplementary table 1 (DOCX 24 kb)

Supplementary figure 1 TDP-43 immunohistochemistry in the deep white matter of the frontal cortex. TDP-43 positive neurites (a, arrow) and oligodendroglial inclusions (b, arrows) are observed in the deep white matter of the frontal cortex. Bar in a represents 20 μm (TIFF 300 kb)
