# Supplementary material for: Homozygosity for the C9orf72 GGGGCC repeat expansion in frontotemporal dementia
Source: Acta Neuropathol. 2013 Jul 2;126(3):401–9. doi: 10.1007/s00401-013-1147-0 (PMC3753468; doi:10.1007/s00401-013-1147-0)
Supplement: Supplementary file 1 — Supplementary table 1 (DOCX 24 kb) [file 401_2013_1147_MOESM1_ESM.docx]

**Supplementary table 1 - Taqman assay primer sequences.** *C9orf72* V1 and V3 were amplified by using transcript-specific forward primers (V1F and V3F respectively) and a common reverse primer (V1+3R) and a common FAM-labelled Taqman probe (V1+3 MGB probe). Variant 2 was amplified using distinct primers (V2F and V2R) and probe (V2 MGB probe).

| **Name** | **Sequence** |
| --- | --- |
| *V1F* | GCGGTGGCGAGTGGATAT |
| *V3F* | GAGCAGGTGTGGGTTTAGGAGA |
| *V1+3R* | TGGGCAAAGAGTCGACATCA |
| *V1+3 MGB probe* | ATTTGGATAATGTGACAGTTGG |
| *V2F* | TCATCTATGAAATCACACAGTGTTC |
| *V2R* | GGTATCTGCTTCATCCAGCTT |
| *V2 MGB probe* | ATGATGATGATATTGGTGAC |
