# Supplementary figures and images for: Homozygosity for the C9orf72 GGGGCC repeat expansion in frontotemporal dementia
Source: Acta Neuropathol. 2013 Jul 2;126(3):401–9. doi: 10.1007/s00401-013-1147-0 (PMC3753468; doi:10.1007/s00401-013-1147-0)

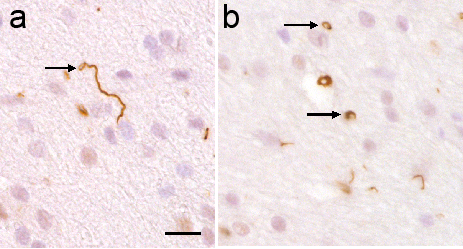

Supplement: Supplementary file 2 — Supplementary figure 1 TDP-43 immunohistochemistry in the deep white matter of the frontal cortex. TDP-43 positive neurites (a, arrow) and oligodendroglial inclusions (b, arrows) are observed in the deep white matter of the frontal cortex. Bar in a represents 20 μm (TIFF 300 kb) [file 401_2013_1147_MOESM2_ESM.tif]
